# “Nano-In-Nano” Schottky Diodes Fabricated by Combining Self-Aligned Nanogap Patterning with Bottom-Up ZnO Nanowire Growth

**DOI:** 10.1021/acsaelm.4c01609

**Published:** 2025-01-02

**Authors:** Umer Farooq Ahmed, Gwenhivir S. Wyatt-Moon, Andrew J. Flewitt

**Affiliations:** Electrical Engineering Division, Engineering Department, University of Cambridge, Cambridge CB3 0FA, U.K.

**Keywords:** ZnO nanowires, Schottky diodes, hydrothermal growth, adhesion lithography, self-assembled monolayer, large-area electronics, nanoelectronics, bottom-up fabrication

## Abstract

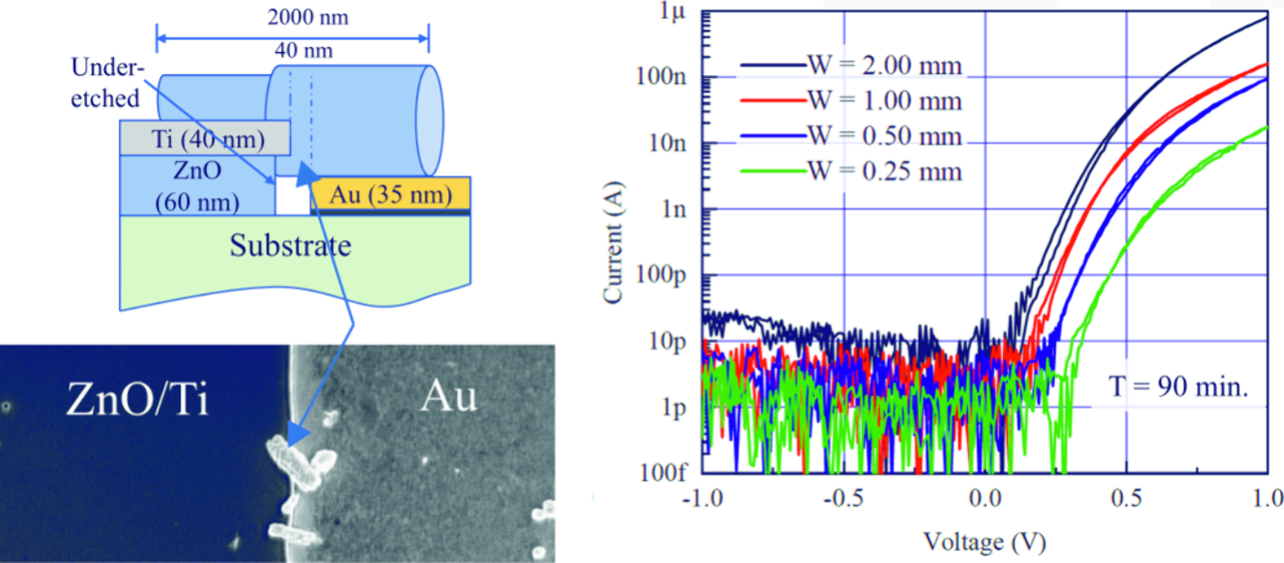

Nanoscale semiconductors offer significant advantages over their bulk semiconductor equivalents for electronic devices as a result of the ability to geometrically tune electronic properties, the absence of internal grain boundaries, and the very low absolute number of defects that are present in such small volumes of material. However, these advantages can only be realized if reliable contacts can be made to the nanoscale semiconductor using a scalable, low-cost process. Although there are many low-cost “bottom-up” techniques for directly growing nanomaterials, the fabrication of contacts at the nanoscale usually requires expensive and slow techniques like e-beam lithography that are also hard to scale to a level of throughput that is required for commercialization. A scalable method of fabricating such devices is demonstrated in this work by combining two bottom-up fabrication techniques. ZnO nanowire Schottky diodes are produced with a device length of a few tens of nanometers and a performance significantly exceeding a ZnO thin film equivalent. The first technique is adhesion lithography that allows self-aligned coplanar electrodes of different materials to be patterned with a nanogap ∼10 to 50 nm length between the two. In this case, one electrode is gold, while the other is a bilayer of titanium on a thin film of ZnO, and it is this thin film that allows the second technique, hydrothermal growth, to be used to grow ZnO nanowires directly across the nanogap. The resulting “nano-in-nano” Schottky diodes have a high rectification ratio >10^4^, a low turn-on voltage <0.3 V, and a minimal off-state current <10 pA. This process could be used to realize a variety of nano-in-nano electronic devices in the future, including short channel gate-all-around (GAA) transistors.

## Introduction

The increase in the computational capability of microprocessors and the ability to store vast quantities of information in small memory devices has enabled the mobile device revolution of the last two decades; this has been achieved by a relentless move to ever smaller fabrication nodes for silicon CMOS fabrication. However, a point is being reached where simple scaling down of transistor channel length to increase system performance is no longer effective. In particular, new device geometries and stacking of devices in three dimensions will be required in future.^1^ In some ways, the introduction of the FINFET structure in mainstream CMOS processing in the early 2000s is an example of this; the traditional “planar” semiconducting channel was replaced with vertically oriented semiconducting fins with a gate wrapped around three sides to improve switching characteristics, but without the need to reduce channel length.^2^ The challenge is to find new ways of fabricating nonplanar device geometries on the nanoscale and to integrate fabrication either as a back-end process onto silicon CMOS or even onto unconventional substrates like glass or plastic for heterogeneous integration in a single package.

This challenge is an opportunity for nanomaterials that can be grown “bottom-up” as a result of crystallographic anisotropy.^3^ Growth techniques like chemical vapor deposition and hydrothermal growth tend to have favored growth directions as a result of crystallographic planes having different surface energies. ZnO, which has a wurtzite structure, is a good example of this, where the polar surface in the *c*-axis [0001] direction is much more reactive than other surfaces and this facilitates the natural growth of nanowires whose long axis is in this direction.^4^ Back-end integration of nanomaterial-based devices becomes possible if growth and subsequent patterning can be achieved at temperatures below previous processing temperatures, and if the entire process can be realized below 250 °C then it starts to become possible to use a diversity of unconventional substrates, like plastics. A nanomaterial can therefore naturally provide the geometry to achieve new device structures. For example, a semiconducting nanowire can provide the basis for a gate-all-around (GAA) transistor structure, which is one possible successor to the FINFET.^5,6^ In addition, the very small volume of nanomaterials results in few defects and an absence of internal grain boundaries with the result that electronic properties close to those of an ideal bulk material might be possible. However, this requires the fabrication of electrical contacts to the nanomaterial, and in order to maintain existing device lengths, the contacts need to be separated by a nanogap of the order of a few tens of nanometers at most. There are few patterning techniques that can achieve such high resolutions, with electron beam lithography (EBL) being one, and although there have been recent developments in automating the process of finding nanomaterials on a surface and using EBL to create a device,^7^ the overall process remains significantly slower and more expensive than that required of production to CMOS standards.

In recent years, a number of techniques have emerged to produce self-aligned metal electrodes on a surface with nanoscale separation. Among these is adhesion lithography (a-lith).^8^ In this process a metal is deposited and patterned on a substrate using basic ultraviolet (UV) optical lithography to form a first electrode. The metal is selectively coated in a self-assembled monolayer (SAM) with the aim of significantly reducing the surface energy. A second metal is then deposited and patterned such that there is an overlap between the two metals along a line where the nanogap between the two coplanar electrodes is required. An adhesive is applied over the entire surface of the substrate which is allowed to dry before being removed. On removal, the adhesive lifts away the second metal only where it overlaps the first because of the presence of the SAM, resulting in the formation of two separate metal electrodes with a nanogap between the two. The length of the gap is a function of the fracture process, which is itself dependent on the thickness of the two metals, the edge profile of the first metal and the microstructure of the second metal. This process can be scaled to large areas,^9^ and it has been demonstrated that electronic devices, such as high frequency Schottky diodes, can be fabricated by depositing a semiconducting thin film into the gap,^10^ or by spin coating a patterned substrate with a solution containing a nanomaterial such that it fills the gap.^11^ The largely dry nature of the a-lith process and its low processing temperature means that it is possible to use unconventional substrates, with diodes having been fabricated on isomalt, for example.^12^

Although the a-lith process can produce nanoscale coplanar electrodes, the issue remains of aligning these with a nanomaterial in an efficient way. It has previously been shown that ZnO nanowires can be grown directly across a macroscale (>5 μm) gap between coplanar electrodes.^13^ This was achieved by sputter-depositing a thin film of ZnO underneath a metal thin film and patterning both layers so that the ZnO is slightly recessed from the overlying metal layer. The ZnO thin film can then seed the hydrothermal growth of lateral ZnO nanowires using the metal layer to prevent vertical (out of plane) growth; the existence of many randomly oriented nanograins of ZnO in the very thin seed layer results in a small number of these being oriented to seed lateral (in plane) nanowire growth between the two electrodes.

This work demonstrates that this approach of seeded lateral growth of ZnO nanowires can be scaled down and effectively combined with a-lith to produce devices on the nanoscale without the need for any high resolution patterning and with the further advantage that ZnO nanowires are only grown where they are needed and so the whole fabrication process is extremely efficient in material use. This has required four key stages of development which are each reported in turn: (1) hydrothermal growth of lateral ZnO nanowires from a ZnO/Ti bilayer, (2) a-lith fabrication of a nanogap between ZnO/Ti and Cr/Au bilayer coplanar electrodes, (3) hydrothermal growth of ZnO nanowires across the nanogap between the coplanar electrodes and (4) electrical testing of the resulting Ti-ZnO nanowire-Au Schottky diodes. Devices are compared with those of the same electrode geometry but with a simple ZnO thin film sputter-deposited into the nanogap.

Although a specific example of integration of the a-lith process with hydrothermal growth of ZnO is presented, the significance of this work is that the same principle could be applied much more widely to straightforwardly produce nanomaterial-in-nanogap (nano-in-nano) electronic devices where the growth of the nanomaterial is directly seeded from a layer underneath either or both electrodes. Nanomaterials that might lend themselves to such fabrication include other nanowires, nanotubes or 2D materials grown by chemical vapor deposition or solution processing techniques.

## Results and Discussion

### ZnO Nanowire Growth from ZnO/Ti Electrodes

**Figure 1:**
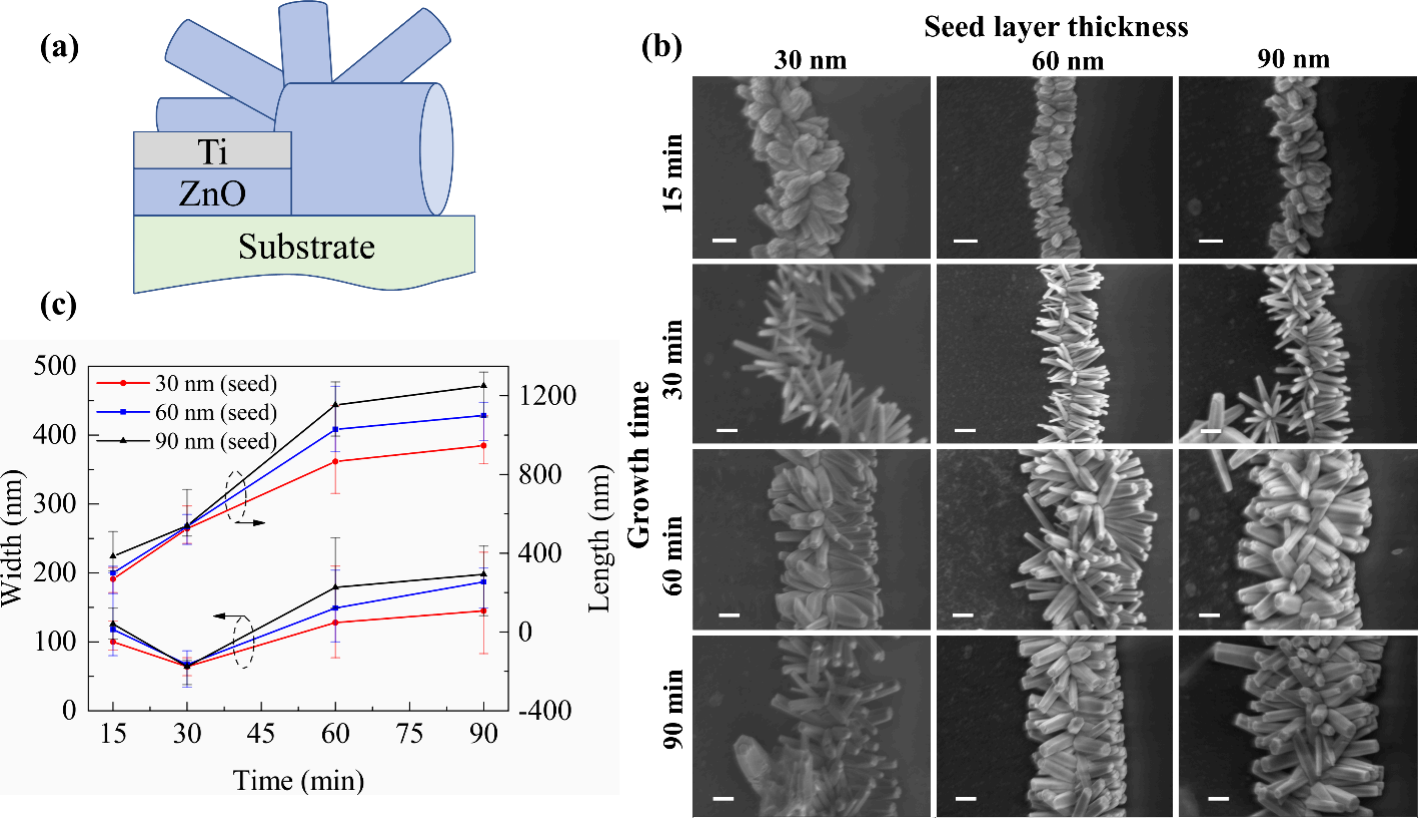
(a) Schematic of the cross section of the ZnO nanowire growth from a Ti-capped ZnO seed layer. (b) SEM images of nanowire morphology as a function of seed layer thickness and growth time (200 nm scale bar and same scale for all images). (c) Quantitative extraction of nanowire length and width as a function of seed layer thickness and growth time.

The key parameters in controlling the lateral growth of ZnO nanowires from a sputter-deposited seed layer underneath a Ti electrode are the thickness of the seed layer, the length of time for the hydrothermal growth (whose general conditions have been previously optimized)^14^ and the edge profile of the ZnO/Ti bilayer. Initially, simple 1.5 mm × 1.0 mm rectangular ZnO/Ti electrodes with a vertical edge profile, as shown in Figure 1, were patterned onto a silicon substrate with a 200 nm SiO_2_ coating. Samples were prepared with ZnO seed layer thicknesses of 30 nm, 60 and 90 nm, and all three thicknesses were exposed to the hydrothermal growth solution for a range of times up to 90 min with the resulting nanowire lengths, widths and overall morphologies measured by scanning electron microscopy (SEM) as shown in Figure 1.

The same basic progression in ZnO nanowire growth is observed for all three ZnO seed layer thicknesses. Initially, growth is laterally away from the ZnO/Ti electrode sidewall with some evidence for a higher longitudinal growth rate during this very initial phase. However, very quickly new ZnO nanowires nucleate in all directions with a progressive decrease in longitudinal growth rate with time. There is a weak influence of ZnO seed layer thickness on longitudinal growth rate, with the thicker seed layer resulting in longer ZnO nanowires for the same growth time. Nanowire width also shows a weak dependence on ZnO seed layer thickness, with thicker seed layers producing wider nanowires, as would be expected. After initial nucleation of ZnO nanowires from the ZnO seed layer, there is little dependence of ZnO nanowire width on growth time.

**Figure 2:**
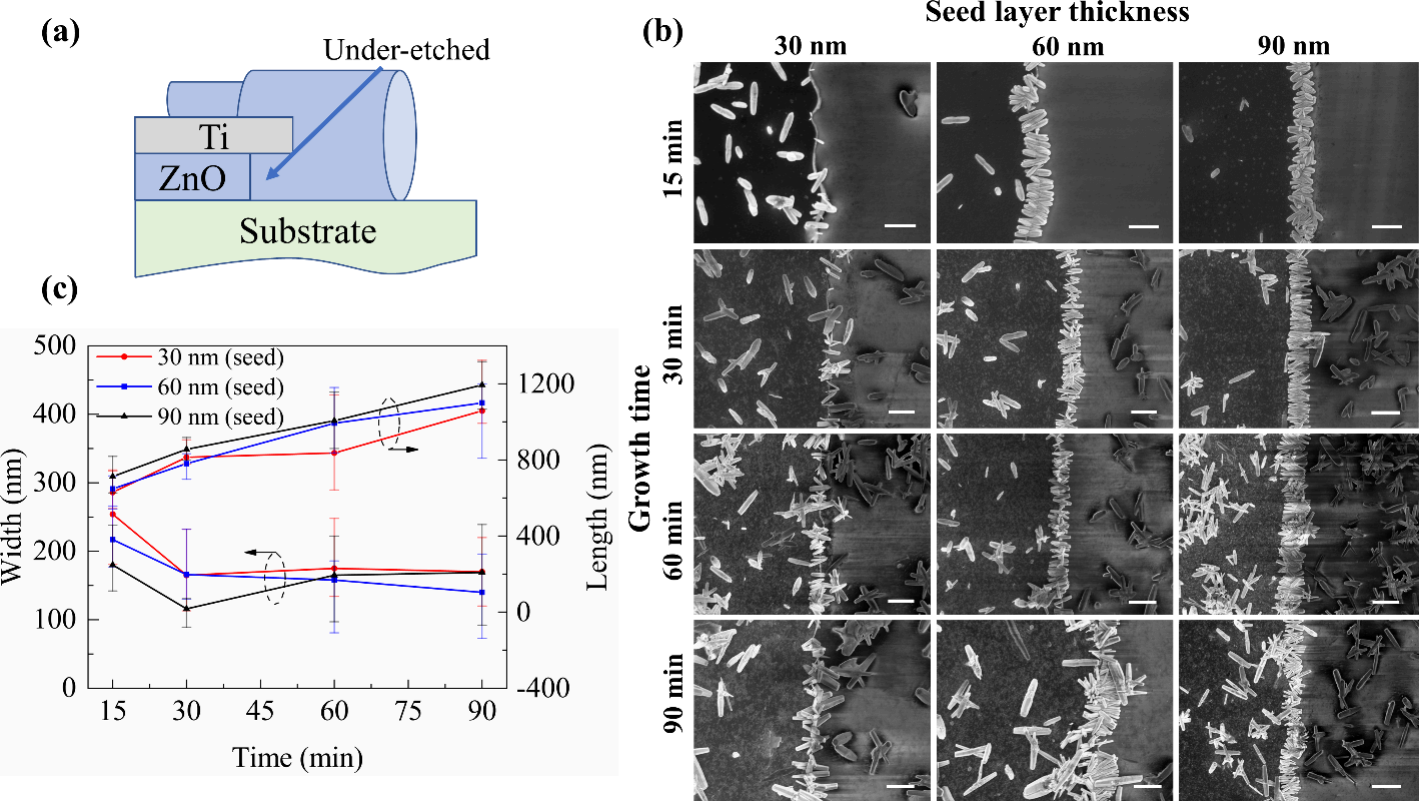
(a) Schematic of the cross section of the ZnO nanowire growth from a Ti-capped recessed ZnO seed layer. (b) SEM images of nanowire morphology as a function of seed layer thickness and growth time (200 nm scale bar and same scale for all images). (c) Quantitative extraction of nanowire length and width as a function of seed layer thickness and growth time.

**Figure 3:**
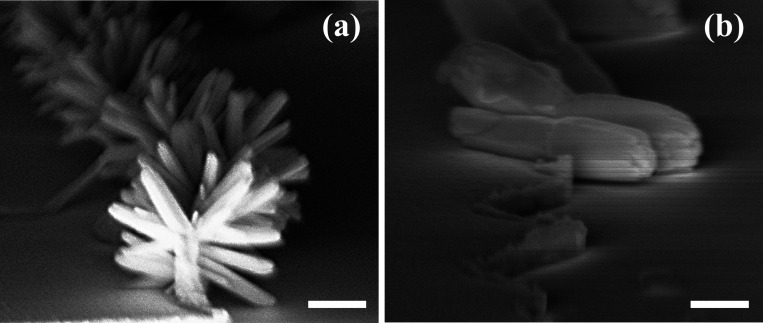
SEM images comparing the morphology of ZnO nanowires after 30 min of hydrothermal growth from (a) a vertical edge profile ZnO/Ti bilayer and (b) a recessed edge profile ZnO/Ti bilayer showing the advantage of the latter structure in producing ZnO nanowires that would connect coplanar electrodes (300 nm scale bar for both images).

Nanowires that do not grow laterally will essentially be parasitic in a nano-in-nano device. Therefore, the same experiment was repeated, but this time with the ZnO seed layer recessed from the edge of the Ti electrode by under-etching in a dilute hydrochloric acid solution prior to hydrothermal growth, as illustrated in Figure 2 along with characterization of the subsequent ZnO nanowire growth. ZnO is so readily etched by HCl that a very dilute solution and a very short etch time is required (see Materials and Methods) such that any impact upon the Ti layer or substrate material is insignificant. The recessed edge profile is observed to significantly limit ZnO nanowire growth to a lateral direction, as desired. There is also a profound effect of ZnO seed layer thickness on the density of nanowires that are nucleated along the electrode edge, with the 30 nm layer resulting in significantly fewer nanowires for every 1 μm of electrode edge. There is less dependence of nanowire length and width on the seed layer thickness. In terms of morphology, ZnO nanowires initially grow laterally away from the electrode and rapidly increase in length and width, with the latter surpassing the thickness of the combined ZnO/Ti bilayer. Once this happens, the ZnO nanowire is able to grow laterally backward over the top of the Ti electrode in addition to continuing to grow laterally away from the electrode, but at a reduced rate and with little further change in width. This is seen most clearly in Figure 3, which compares the morphology of ZnO nanowires produced from the vertical and recessed electrode edge profiles. For the purposes of fabricating nano-in-nano devices, the recessed edge structure is clearly advantageous.

It is interesting to note that the nanowires grow preferentially in a direction perpendicular to the edge of the Ti electrode, which from a device fabrication perspective is a favorable situation. There are two probable explanations for this. The first is that the HCl etches the ZnO seed layer isotropically, and so a vertical side wall of ZnO is produced parallel to the edge of the Ti electrode. Only where this wall has a highly reactive (0001) surface will nanowire growth be nucleated in the same orientation. Second, there may be some steric hindrance effect with the perpendicular growth direction resulting in the greatest nanowire packing density.

Detached ZnO nanowires are also clearly visible on the sample surfaces in Figure 2. These samples were prepared without any attempt to remove loose ZnO nanowires, such as through ultrasonication.

### A-lith Fabrication of Nanogaps between Recessed ZnO/Ti and Cr/Au Electrodes

**Figure 4:**
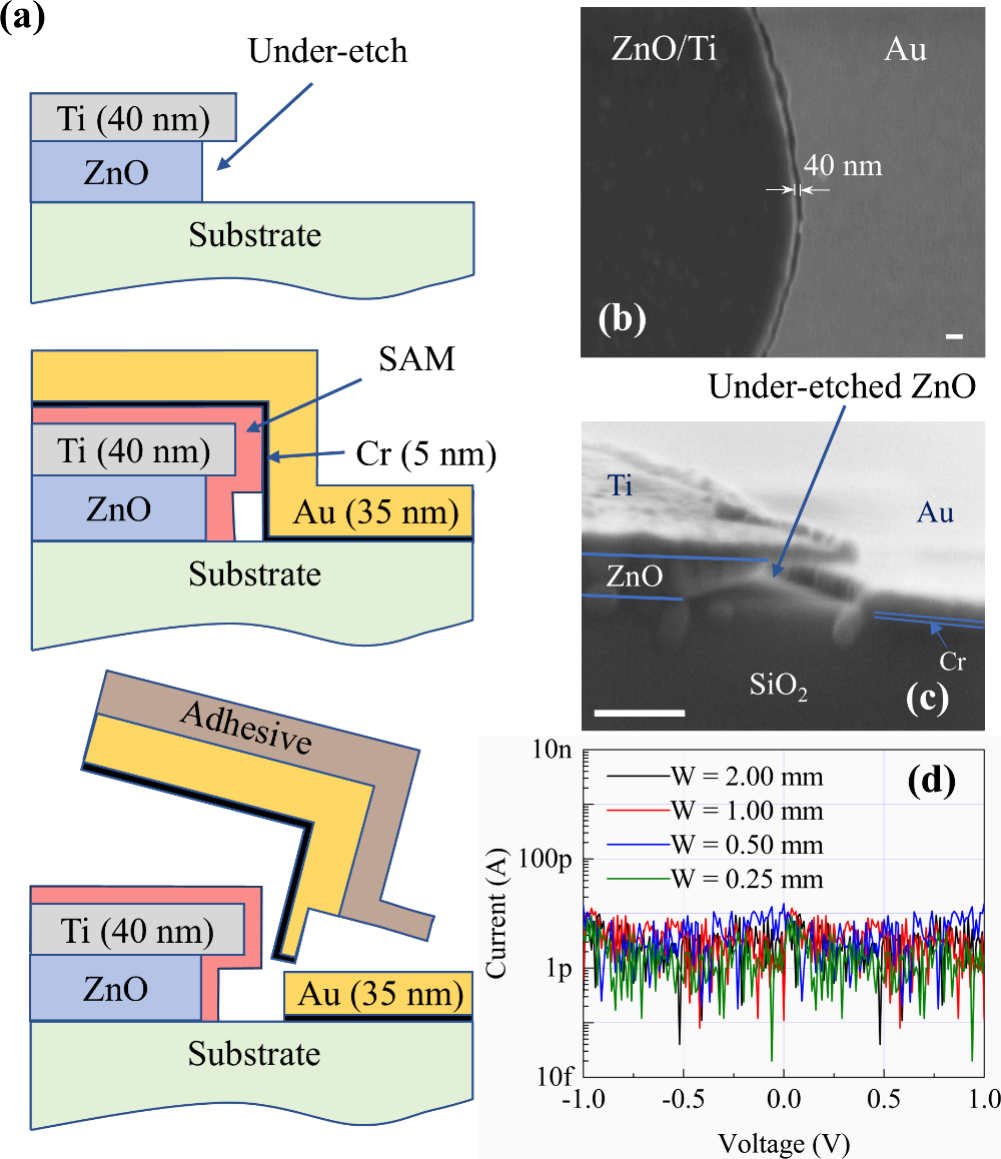
(a) Schematic diagram of the a-lith process. (b) SEM image of the nanogap created between ZnO/Ti and Cr/Au coplanar electrodes by the a-lith process. (c) Cross section SEM image of the nanogap (100 nm scale bar for both (b) and (c)). (d) Electrical characteristics of the nanogap showing electrical isolation.

The a-lith process employed for two simple metal electrodes relies on the use of the SAM to reduce the surface energy between the two metals relative to the surface energy between each metal and the substrate and that between the second metal and the adhesive so that the second metal is selectively removed where it overlaps the first. A 5 nm Cr adhesion layer was successfully employed to ensure that the 35 nm layer Au adhered sufficiently to the substrate to avoid undesired removal.^10^ However, the use of a recessed ZnO seed layer underneath the 40 nm Ti layer as an a-lith electrode has not been previously reported. A schematic diagram of the whole process is shown in Figure 4(a).

The ZnO/Ti bilayer was always used as the first electrode in the a-lith process. The bilayer was produced in the same way as for the previous section but with either a 30 or 60 nm thick ZnO layer only (90 nm was not used). An HCl under-etch was performed to create the recessed edge structure before the sample was exposed to an O_2_ plasma which was found to improve the subsequent attachment of the octadecylphosphonic acid (ODPA) SAM as it is known that this molecule attached particularly well to slightly oxidized metal surfaces.^8^ The a-lith process then proceeded with deposition and patterning of the Cr/Au bilayer as the second electrode and adhesive-based removal of any overlapping electrode materials.

Figure 4(c) shows a cross-section SEM image of a typical coplanar electrode structure with a nanogap. Critically, the recessed edge structure of the ZnO/Ti electrode is preserved in the a-lith process. A consistent gap of ∼40 nm length is produced between the coplanar electrodes. Structures with widths varying from 250 μm up to 2.0 mm were produced. Current–voltage (I–V) measurements on these structures, shown in Figure 4(d), confirm that they were electrically isolated with currents below 10 pA, which is around the noise floor of the electrical test system used.

### ZnO Nanowire Growth across the Nanogap

**Figure 5:**
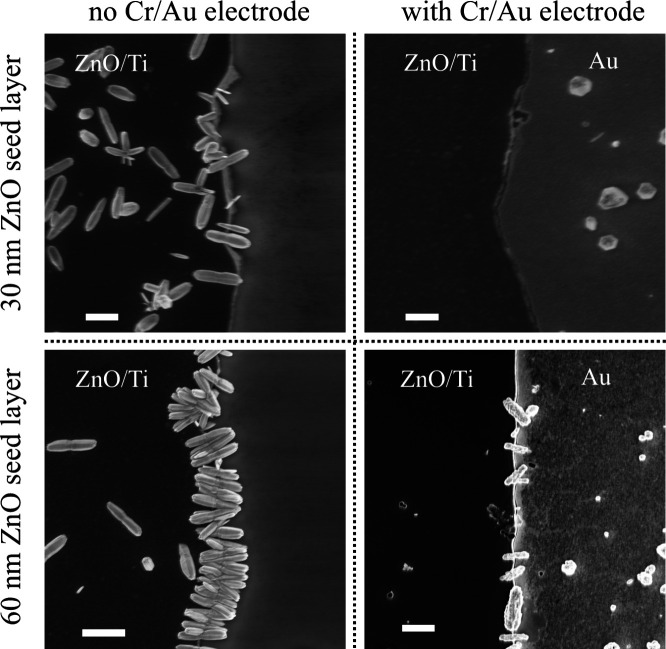
SEM images after 60 min of hydrothermal ZnO nanowire growth (left) without and (right) with a Cr/Au electrode present for a (top) 30 nm and (bottom) 60 nm ZnO seed layer showing that the Cr/Au reduces the effective thickness of the ZnO seed layer for the purposes of nanowire growth (300 nm scale bar for all images).

Coplanar electrode structures with both a 30 and 60 nm ZnO seed layer were subjected to the hydrothermal growth process for ZnO nanowires with the aim of growing them directly from the ZnO/Ti electrode to the Cr/Au electrode across the nanogap. In both cases, samples had regions where there was only a ZnO/Ti electrode with no Cr/Au electrode close by to act as a control. As seen in the resulting SEM images of Figure 5, the thickness of the ZnO seed layer relative to that of the combined Cr/Au electrode was found to be critical. When the ZnO seed layer was thinner (30 nm) than the combined Cr/Au electrode (40 nm), no hydrothermal ZnO nanowire growth was observed. Only when the ZnO seed layer was thicker (60 nm) than the Cr/Au electrode so that the edge of the ZnO seed layer was clearly exposed to the growth fluid was nanowire growth observed, and even then at a rather lower density (per unit electrode width) than for the control without the Cr/Au electrode nearby. The effective thickness of the ZnO seed layer for nanowire growth is the physical ZnO seed layer thickness less the thickness of the other electrode. Where the effective thickness of the ZnO layer is sufficient, ZnO nanowires appear to grow laterally across the nanogap, and lateral growth in the reverse direction is also observed, as found when there was no Cr/Au electrode nearby.

### Ti-ZnO Nanowire-Au Schottky Diodes

**Figure 6:**
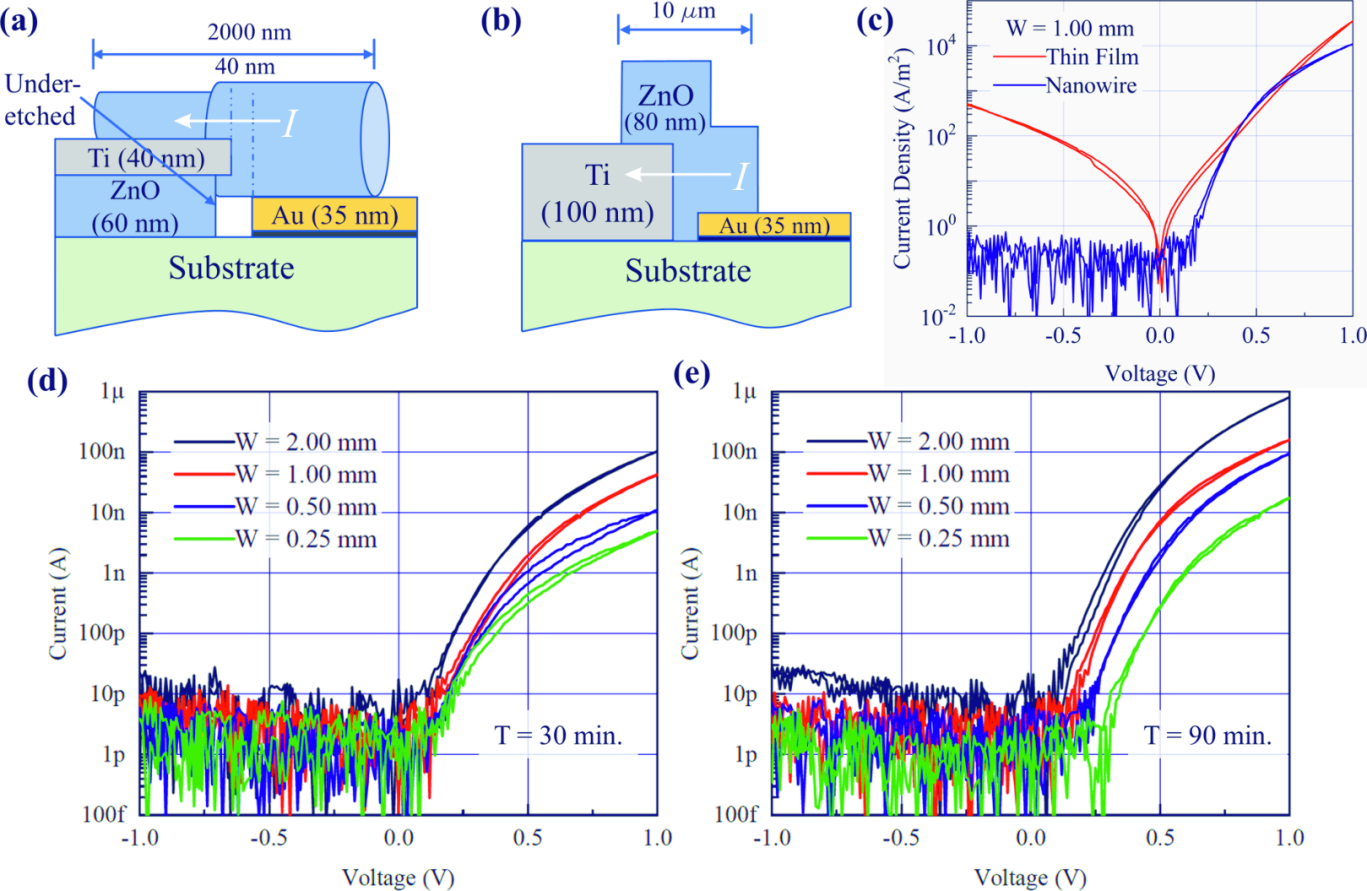
Schematic diagram of the (a) ZnO nanowire and (b) ZnO thin film Schottky diodes where the direction of classical current flow is indicated by the arrow. (c) comparison of I–V characteristics between the nanowire and thin film devices. I–V characteristics for different ZnO nanowire device widths after (d) 30 min of hydrothermal growth and (e) 90 min of hydrothermal growth.

The devices fabricated with a 60 nm ZnO seed layer were electrically tested and hydrothermal growth times of 30 and 90 min were utilized, the results of which are shown in Figure 6, for device widths ranging from 250 μm to 2.0 mm where the bias is applied to the Au contact and the Ti contact is held at 0 V. The Au is expected to form a Schottky junction with ZnO whereas Ti is expected to form an Ohmic contact. Therefore, the complete Ti-ZnO nanowire-Au structure should behave as a Schottky diode, and this is observed for all devices. Under reverse bias, the current flow is at or close to the noise floor of the measurement system, being below 10 pA for almost all device widths and both growth times. Forward bias results in a significant current flow once a turn-on threshold voltage is reached, which is typically in the range from 0.1 to 0.3 V. There is a clear dependence of the turn-on voltage with device width with devices of 250 μm width having a higher turn-on voltage than wider devices. This effect is more pronounced for the devices fabricated with a 90 min hydrothermal growth time compared with a 30 min time. As a result, the on-state current at a forward bias of 1.0 V scales superlinearly with device width, as wider devices not only have a greater number of nanowires acting as conduction paths, but they also have a lower turn-on voltage. Increasing the hydrothermal growth time increases the number of nanowires per unit width, as shown in Figure 5, and this results in a greater current for devices fabricated with a 90 min hydrothermal growth time. Given that the off-state current does not scale significantly with device width, the 2.0 mm wide devices fabricated with this longer hydrothermal growth time exhibit the greatest switching ratio of 10^5^ at ±1 V.

For comparison devices based on a sputter-deposited thin film of ZnO between coplanar electrodes of Ti and Cr/Au were also fabricated using the a-lith process. The cross-section of both structures is shown in Figure 6, but the key difference for the thin film devices are that the bilayer of 60 nm ZnO and 40 nm Ti is replaced with a single layer of 100 nm Ti to maintain the same overall electrode thickness so that the geometry of the nanogap produced when the Cr/Au material is removed by the adhesive is the same as for the ZnO nanowire devices. The electrical characteristics of devices with a width of 1.0 mm are shown and in the case of the Ti-ZnO nanowire-Au device, the device fabricated with a 90 nm hydrothermal growth time is shown. In both cases, the current is scaled per unit width of the device to allow straightforward comparison. The advantage of the Ti-ZnO nanowire-Au device over the Ti-ZnO thin film-Au device is immediately apparent, with the latter having a significantly greater off-state current. Interestingly, the on-state current densities are very similar in spite of the actual cross section of conducting material being much greater in the thin film device. This indicates significantly better conduction in the ZnO nanowires as would be expected given the lower defect density as a result of the absence of grain boundaries in the nanowire, but which will be present in a thin film. Grain boundaries are rich in defects which act as traps and scattering centers and are known to have a deleterious effect on conduction.^15^ Their absence in the ZnO nanowires is therefore a significant benefit.

## Conclusions

Nanomaterials like nanowires, nanotubes and 2D materials offer a route to continue to advance the power of electronics but without having to rely on physical scaling, which has underpinned much of the Moore’s law progress of the last five decades. This is achieved through both geometrical and electronic conduction advantages that these nanomaterials possess. However, practical integration of these into a complete system is a major challenge. Although “bottom-up” approaches to nanomaterial growth are generally environmentally more efficient than “top-down” methods, the reality is that complete systems will require multiple bottom-up fabrication steps. It is shown that two complementary bottom-up processes can be combined successfully–a-lith for nanogap coplanar metal electrode fabrication and seeded hydrothermal growth of ZnO nanowires–to realize complete Schottky diode devices from ZnO nanowires that are grown directly between ZnO/Ti and Cr/Au coplanar electrodes separated by a gap ∼40 nm. Fabrication is achieved using only low cost, scalable processing at low temperatures (the maximum of 110 °C being associated with photoresist baking). The ZnO nanowire Schottky diodes have characteristics that significantly outperform their thin film equivalents, namely a turn-on voltage <0.3 V, a switching ratio at ±1 V of up to 10^5^ and a very low off-state current typically below 10 pA. This is because the nanogap between the electrodes allows the nanoscale properties of the nanowires to be properly utilized. Therefore, the fundamental principle demonstrated in this work is that the combination of bottom-up nanoscale patterning combined with bottom-up nanomaterial growth to create nano-in-nano devices could be applied much more widely to other device geometries and nanomaterial systems.

## Materials and Methods

### Substrate and Electrode Deposition

Substrates used for this work were 20 × 20 mm squares of p-doped Si wafers coated with (200 ± 20) nm of thermally grown SiO_2_. The substrate was cleaned in an ultrasonic bath of acetone followed by IPA for 10 min before being blow dried under dry nitrogen. The ZnO seed layer (thickness as described in the main text) was deposited by reactive RF (13.56 MHz) magnetron sputtering (CCR Technologies GmbH) at 75 W using a Zn target (99.9% purity) in an O_2_ rich environment with a gas flow of 25 sccm of Ar and 18 sccm of O_2_ held at a pressure of 8 × 10^–3^ mbar. Ti (99.9% purity) was deposited on top of the ZnO seed layer (40 nm thickness unless stated) by DC magnetron sputtering at 100 W in a 30 sccm gas flow of Ar held at a pressure of 3.5 × 10^–3^ mbar. Cr (5 nm thickness) and Au (35 nm thickness) (both 99.9% purity) were thermally evaporated (Edwards E306A) at a rate of ∼0.1 nm s^–1^ with a base pressure prior to deposition of 1 × 10^–6^ mbar.

### A-lith Process

A photolithographic lift-off process was used to pattern the electrode materials used in the a-lith fabrication. AZ5214E photoresist was spin coated at 4000 rpm for 40 s prior to baking at 100 °C for 60 s on a hot plate. Exposure was carried out on an EVG620 mask aligner with a mercury lamp (365 nm, 10 mW cm^–2^) for 7 s through a chrome plated quartz mask with 2.25 × 1 mm, 1.25 × 1 mm, 0.75 × 1 mm and 0.50 × 1 mm rectangles. A postbake was then carried out on a hot plate at 110 °C for 120 s before an inversion flood exposure for 14 s on the mask aligner with otherwise the same conditions as for the initial exposure. Finally, development was carried out in a 1:4 solution of AZ351B:DI water for 20 s. Development was quenched in DI water. An acetone bath followed by an IPA rinse was used for lift-off.

After patterning, the Ti surface was treated with an O_2_ plasma (ZEPTO B, Diener Electronics) for 150 s at 100 W in a 5 sccm gas flow of pure O_2_ held at a pressure of 1.0 mbar to aid SAM attachment. The SAM coating solution was prepared by mixing 50 mg of ODPA in 30 mL IPA (99.9% purity) and stirring. The sample was then placed upside down in the solution for 4 h. This was followed with an IPA rinse. Samples were dried with dry nitrogen and placed on a hot plate at 70 °C for 120 s.

The same mask was used for lift-off patterning of the Cr/Au electrodes, but with an offset to give a 0.5 mm overlap of materials. A-lith selective removal of the overlapping Cr/Au was performed by brush coating the sample in First Contact Polymer Glue (Photonic Cleaning Technologies Inc.). The glue was allowed to dry in ambient clean room conditions for 2 h before peeling removal with tweezers. Remaining ODPA was removed by O_2_ plasma etching for 120 s using otherwise the same conditions as for the Ti surface treatment.

### Seed Layer under Etching and ZnO Nanowire Growth

The ZnO seed layer was under etched in a highly dilute (1:5000) solution of HCl in DI water for 5 s. Hydrothermal growth of ZnO nanowires was performed using a 0.025 M solution of hexamethyltetramine (HMTA) and zinc nitrate dihydrate in DI water at 80 °C. Loose nanowires were removed after hydrothermal growth by ultrasonication in IPA for 60 s.

### Characterization

SEM was carried out using a Zeiss 1530VP (Carl Zeiss Microscopy Ltd.) operating at 3 kV. Electrical characterization was performed on a Cascade probe station (Cascade Microtech Ltd.) using an Agilent B1500A semiconductor parameter analyzer.

## Notes

For the purpose of open access, the author has applied a Creative Commons Attribution (CC BY) license to any Author Accepted Manuscript version arising from this submission. Raw data for this work may be downloaded from the University of Cambridge repository at https://www.repository.cam.ac.uk/home.

## ABBREVIATIONS

a-lith: adhesion lithogtaphy
CMOS: complementary metal oxide semiconductor
GAA: gate-all-around; HMTA, hexamethyltetramine
I–V: current–voltage
ODPA: octadecylphosphonic acid
SAM: self-assembled monolayer
SEM: scanning electron microscopy

## References

[ref1] Lundstrom M. S.; Alam M. A. Moore’s law: The journey ahead. Science 2022, 378 (6621), 722–723. 10.1126/science.ade2191. 36395227

[ref2] Bohr M. The Evolution of Scaling from the Homogeneous Era to the Heterogeneous Era. In 2011 International Electron Devices Meeting; IEEE: Washington, DC, 2011, pp 1.1.1–1.1.6 10.1109/IEDM.2011.6131469.

[ref3] Baig N.; Kammakakam I.; Falath W. Nanomaterials: a review of synthesis methods, properties, recent progress, and challenges. Mater. Adv. 2021, 2 (6), 1821–1871. 10.1039/D0MA00807A.

[ref4] Unalan H. E.; Hiralal P.; Rupesinghe N.; Dalal S.; Milne W. I.; Amaratunga G. A. J. Rapid synthesis of aligned zinc oxide nanowires. Nanotechnology 2008, 19 (25), 255608 10.1088/0957-4484/19/25/255608. 21828660

[ref5] Das R. R.; Rajalekshmi T. R.; James A. FinFET to GAA MBCFET: A Review and Insights. IEEE Access 2024, 12, 50556–50577. 10.1109/ACCESS.2024.3384428.

[ref6] Pandey A. Recent Trends in Novel Semiconductor Devices. Silicon 2022, 14 (15), 9211–9222. 10.1007/s12633-022-01694-8.

[ref7] Potocnik T.; Christopher P. J.; Mouthaan R.; Albrow-Owen T.; Burton O. J.; Jagadish C.; Tan H. H.; Wilkinson T. D.; Hofmann S.; Joyce H. J.; Alexander-Webber J. A. Automated Computer Vision-Enabled Manufacturing of Nanowire Devices. ACS Nano 2022, 16 (11), 18009–18017. 10.1021/acsnano.2c08187. 36162100 PMC9706672

[ref8] Beesley D. J.; Semple J.; Jagadamma L. K.; Amassian A.; McLachlan M. A.; Anthopoulos T. D.; Demello J. C. Sub-15-nm patterning of asymmetric metal electrodes and devices by adhesion lithography. Nat. Commun. 2014, 5, 3933 10.1038/ncomms4933. 24861953 PMC4050269

[ref9] Wyatt-Moon G.; Flewitt A. J. Adhesion Lithography Peel Tool Design; Apollo - University of Cambridge Repository: 2021 10.17863/CAM.68204.

[ref10] Wyatt-Moon G.; Niang K. M.; Rider C. B.; Flewitt A. J. Air stable Indium-Gallium-Zinc-Oxide diodes with a 6.4 GHz extrinsic cut-off frequency fabricated using adhesion lithography. IEEE Electron Device Lett. 2020, 41 (1), 175–178. 10.1109/LED.2019.2953982.

[ref11] Georgiadou D. G.; Semple J.; Sagade A. A.; Forstén H.; Rantakari P.; Lin Y.-H.; Alkhalil F.; Seitkhan A.; Loganathan K.; Faber H.; Anthopoulos T. D. 100 GHz zinc oxide Schottky diodes processed from solution on a wafer scale. Nat. Electron. 2020, 3, 718–725. 10.1038/s41928-020-00484-7.

[ref12] Wyatt-Moon G.; Saravanavel G.; Sambandan S.; Flewitt A. J. Nanodiodes on a Digestible Substrate. IEEE Electron Device Lett. 2023, 44 (2), 337–340. 10.1109/LED.2022.3231080.

[ref13] Swanwick M. E.; Pfaendler S. M.-L.; Akinwande A. I.; Flewitt A. J. Near-ultraviolet zinc oxide nanowire sensor using low temperature hydrothermal growth. Nanotechnology 2012, 23, 344009 10.1088/0957-4484/23/34/344009. 22885284

[ref14] Chevalier-César C.; Capochichi-Gnambodoe M.; Leprince-Wang Y. Growth mechanism studies of ZnO nanowire arrays via hydrothermal method. Applied Physics A-Materials Science & Processing 2014, 115 (3), 953–960. 10.1007/s00339-013-7908-8.

[ref15] Seto J. Y. W. Electrical properties of polycrystalline silicon films. J. Appl. Phys. 1975, 46 (12), 5247–5254. 10.1063/1.321593.

